# Female Representation: Australian Diabetes and Endocrinology Societies

**DOI:** 10.3389/fendo.2022.842909

**Published:** 2022-03-10

**Authors:** Lisa M. Raven, Ann I. McCormack

**Affiliations:** ^1^Department of Endocrinology, St. Vincent’s Hospital, Sydney, NSW, Australia; ^2^St. Vincent’s Clinical School, Faculty of Medicine, The University of New South Wales, Sydney, NSW, Australia; ^3^Garvan Institute of Medical Research, Sydney, NSW, Australia

**Keywords:** gender, female, endocrinology, plenary speaker, medical society

## Abstract

**Background:**

Endocrinology has one of the highest proportions of female specialists and trainees, however females have traditionally been underrepresented in leadership positions and as speakers at scientific meetings.

**Hypothesis:**

Females would represent less than half of invited speakers (plenary, symposium sessions) at endocrinology conferences and in leadership positions of endocrinology societies.

**Method:**

An audit of Australian diabetes and endocrinology societies and their respective annual scientific meetings between 2016 – 2020. Analysis of the gender of conference speakers across oral, symposium and plenary sessions, session chairs, program organising committees and society committees.

**Results:**

A total of 1638 speakers (females 856, 52.3%) across 550.4 hours (females 273.6, 49.7%) of presentations at the conferences were identified. Among plenary sessions of all 3 societies there were more male (61%) than female speakers. A total of 608 session chairs were identified, with 313 (51.5%) females. The majority of organising committee members (n=116) were female (56%), however the representation across each organising committee varied. There was a low proportion of society female council members (39% female).

**Conclusion:**

There was an equal representation of females and males as conference speakers and session chairs. However, there was an underrepresentation of women in more prestigious roles of plenary speakers and society council members. We implore conscious efforts to address this disparity.

## Introduction

The proportion of female doctors is increasing, however the representation of females in leadership positions of scientific societies and academia is lagging and females have been underrepresented as presenters at medical conferences (1–6). Of physician specialities, endocrinology has one of the highest proportions of female workforce, with females representing 55% of consultant endocrinologists in Australia (7). Similarly in Europe and Canada the majority of endocrinologists are female, 56% and 61% respectively (1). Despite this, there is underrepresentation of women at endocrinology conferences, on society boards, in authorship of endocrinology guidelines and editorial positions of scientific journals (1, 6, 8–10). The reasons for this are not entirely understood but barriers to females advancing through academia and into leadership positions are well documented in healthcare and require multi-faceted organisational intervention to address such disparities (11). There is no publicly available data on the rates of academic versus clinical endocrinologists in Australia and the female representation among academic endocrinologists (physician with university appointment) in Australia. However, recent meta-analytic data demonstrates the continued existence globally of a significant gender gap in academic medicine across all specialities (5).

We hypothesised that females would represent less than half of invited speakers (plenary and symposium sessions) at endocrinology conferences and in leadership positions of endocrinology societies. To assess gender representation across meetings and leadership positions within Australian diabetes and endocrinology societies, we conducted an audit of endocrinology conference programs from 2016 to 2020, assessing gender of oral, symposium and plenary speakers, session chairs, program organising and society committees (council and office bearers being president, president-elect, treasurer and secretary).

## Method

The three major adult Australian diabetes and endocrinology societies, and their respective annual scientific meetings, were identified as the Australian Diabetes Society (ADS; Australasian Diabetes Congress), Australian and New Zealand Bone and Mineral Society (ANZBMS; ANZBMS Annual Scientific Meeting) and the Endocrine Society of Australia (ESA; ESA Annual Scientific Meeting). An internet search identified the programs from each of these meetings between the years 2016-2020. From each conference program we extracted the first and last name of speakers, session chairs, program and local organising committee members and society office bearers for the corresponding years. All conferences had program organising committees, and some conferences additionally had local organising committees, members of both of these committees were included and referred to as organising committees. We assigned a binary definition of gender (man or woman) based on publicly available information assessed through online searches. Each session was categorised as either oral, symposium or plenary sessions as indicated in the conference program. The amount of time of each presentation was recorded. Sponsored breakfast and lunch symposiums were excluded. Speakers who presented more than once within a single session were counted as many times as they presented. Where there were 2 presenters listed, the time was distributed equally, and they were listed as separate presenters. Where conferences were combined and there was a joint session allocated to both conferences, the session was recorded in both the respective conference data. Descriptive statistics were used to evaluate the proportion of female presentation time, chairpersons, committee and society council members. The proportion of presentation time was assessed to reflect the hierarchy of plenary and symposium presentations that traditionally are longer than oral presentations. Data were expressed as mean of raw data +/- standard deviation or as number and percentages. Formal ethics approval was not required for this research as only publicly available information was reported.

## Results

In total 17 conferences were included, with one conference (2016 ESA-SRB-ANZBMS Annual Scientific Meeting) having the sessions split into the respective society data. We identified a total of 1638 speakers across 550.4 hours of presentations at the conferences, with 856 female (F) speakers (52.3%) and 273.6 hours (49.7%) of female presentations. It should be noted that the hours of presentations at the 2020 ESA and ANZBMS scientific meetings were reduced compared to other years, in the context of being virtual conferences during the SARS-CoV-2 pandemic.

**Figure 1:**
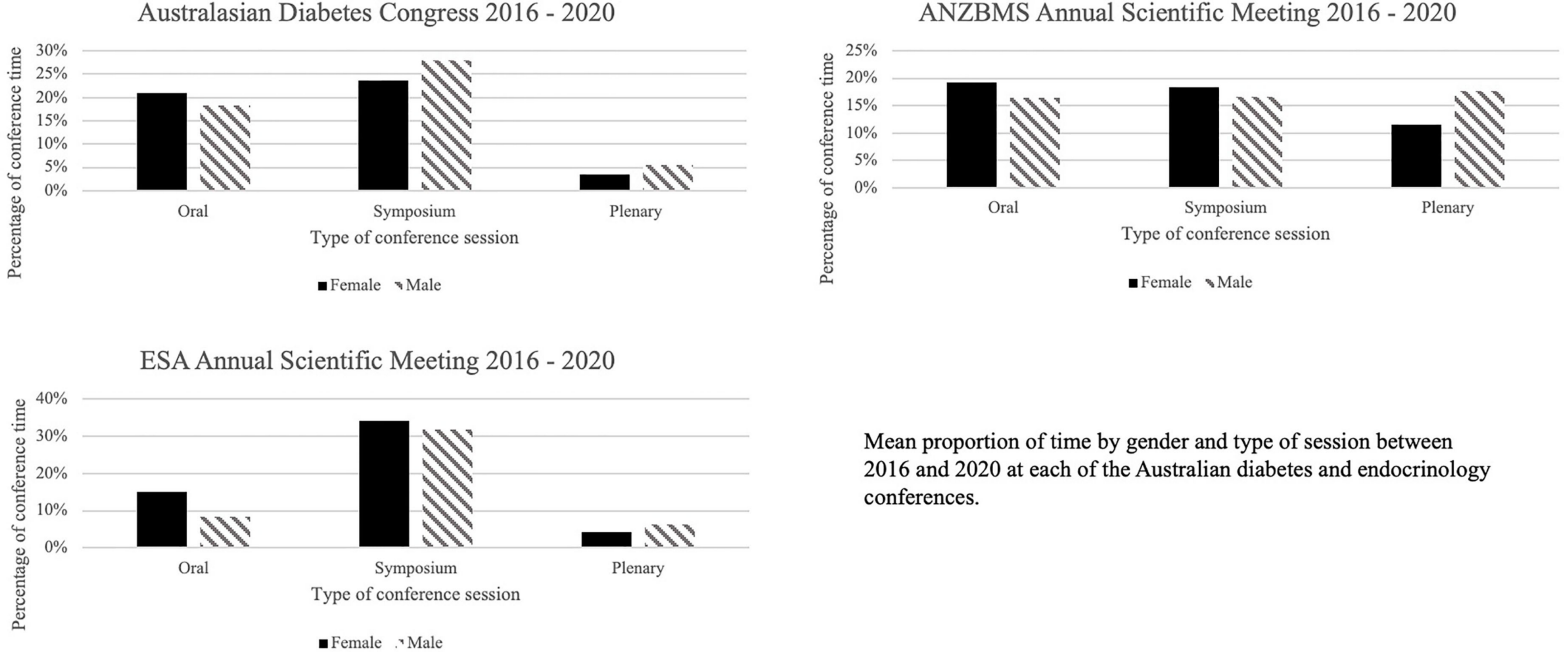
Proportion of conference presentation time by gender and type of session. ANZBMS, Australian and New Zealand Bone and Mineral Society; ESA, Endocrine Society of Australia.

Overall oral presentations accounted for 35% of conference time (total of 192.2 hours), symposium sessions accounted for 51% (283.4 hours) and plenary sessions accounted for 14% (74.8 hours). There was a predominance of female speakers in oral presentations at the ESA conferences (65%), with females representing just over half of oral presentations at ADS and ANZBMS (53% and 54% respectively) (**Figure 1**). Across symposium sessions, females accounted for a fraction more than half of speakers at the ESA and ANZBMS conferences (52% and 53% respectively), while at the ADS they represented slightly less than half (46%). Among plenary sessions of all 3 societies there was more male (M) than female presentation time (ADS 61% M, ANZBMS 60% M, ESA 59% M). Overall, there was an increase in the proportion of female presentation time in symposiums and plenary sessions from 2016 to 2020.

**Figure 2:**
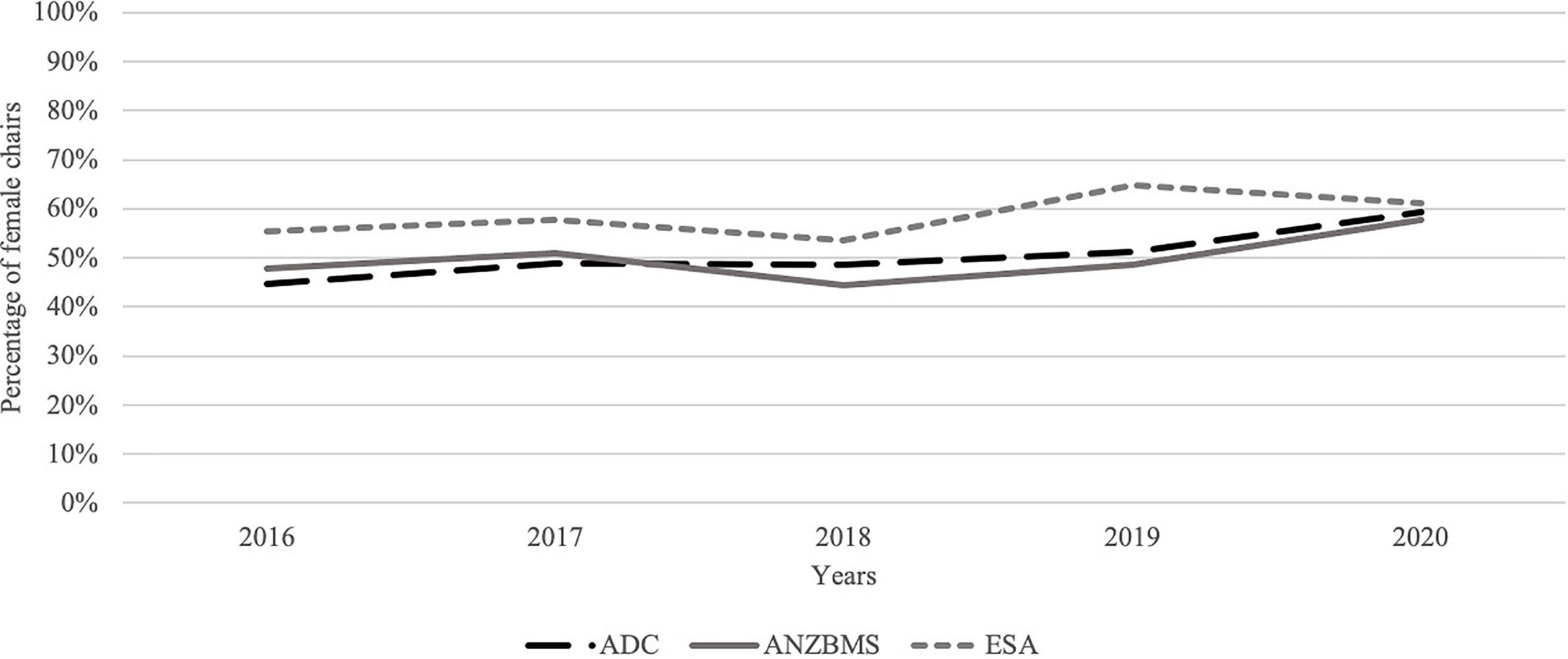
Percentage of female session chairs. ADC, Australasian Diabetes Congress; ANZBMS, Australian and New Zealand Bone and Mineral Society; ESA, Endocrine Society of Australia.

A total of 608 session chairs were identified, with 313 (51.5%) females. There was an increase in the proportion of female chairs from 2016 to 2020 (**Figure 2**).

**Figure 3:**
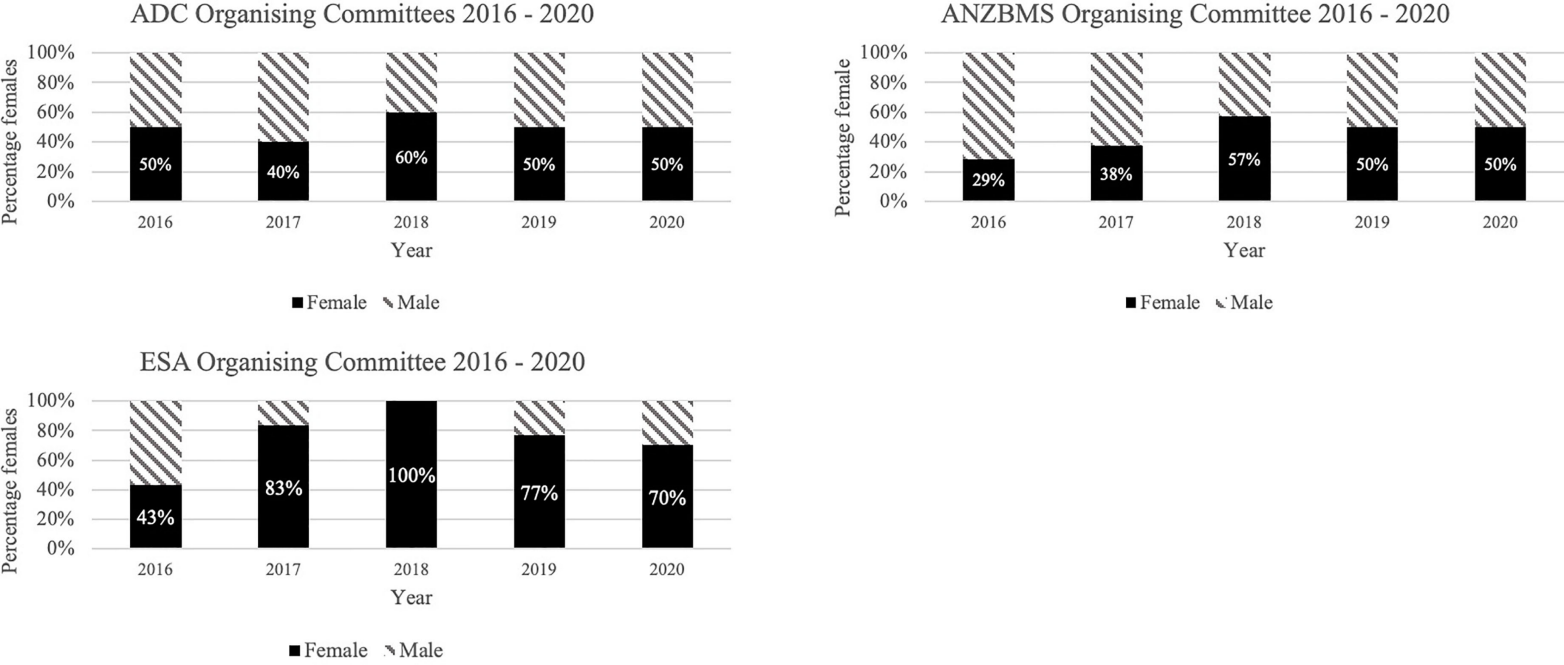
Proportion of conference organising committee by gender. ADC, Australasian Diabetes Congress; ANZBMS, Australian and New Zealand Bone and Mineral Society; ESA, Endocrine Society of Australia.

Conference organising committees were identified for all conferences. The number of organising committee members varied, with a mean ± SD of 7.8±4.3. Overall, the majority of organising committee members (n=116) were female (56%), however the representation on each organising committee varied (**Figure 3**).

**Figure 4:**
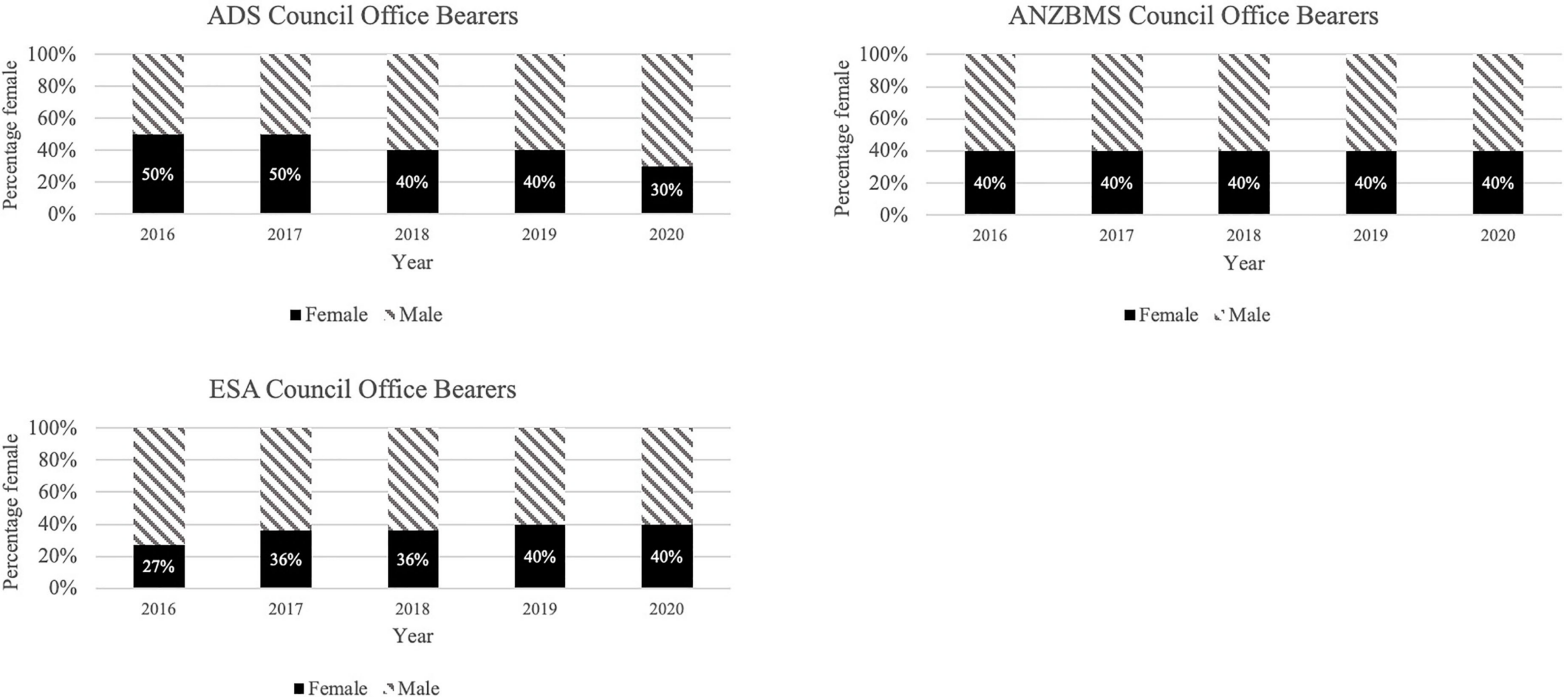
Proportion of society council members by gender. ADS, Australian Diabetes Society; ANZBMS, Australian and New Zealand Bone and Mineral Society; ESA, Endocrine Society of Australia.

We identified a total of 153 council members across all societies for the years 2016-2020, with 39% female. Each society had between 10 and 11 officer bearers each year. There was a downward trend in female representation on ADS council (50% in 2016 *vs* 30% in 2020), an upward trend in female officer bearers on ESA council (27% in 2016 *vs* 40% in 2020) while females continued to represent 40% of positions across these years on ANZBMS council (**Figure 4**). However, the proportion of more senior positions (office bearers) on both ESA and ANZBMS councils remained at 25% between 2017 and 2020.

## Discussion

Overall females were equally represented among all speakers (oral, symposium, plenary) at endocrinology conferences in Australia between 2016-2020. However, females were underrepresented as speakers in plenary sessions and as society council members and office bearers.

In Australia, the majority of endocrinologists and endocrinology trainees are female. Data from 2015-2017 reported that between 49-55% of consultant endocrinologists in Australia were female, which was the highest representation of females in any physician specialty (7, 12, 13). Internationally it is anticipated that the proportion of female endocrinologists will continue to increase (4). In Australia the average age of female endocrinologists is 7.7 years younger than the average age of male endocrinologists, and in 2016 the vast majority (80%) of endocrinology trainees were female (13).

We found a similar gender distribution of conference speakers (52.3% F overall) to the gender proportion of endocrinologists in our region. However, we identified a male predominance (61%) among plenary speakers across all 3 societies. This would support the findings of Arora et al. who reported underrepresentation of females as invited speakers at academic medical conferences across multiple specialities and countries, as well as Gamage et al. who more specifically reported underrepresentation of females as invited speakers at endocrinology conferences in North America and Europe (1, 6).

We noted with interested the findings of Arora et al. that females represented just 28% of invited speakers and panellists at the 2018 Annual ESA Seminar, which is a separate meeting to the ESA Annual Scientific Meeting (1). For comparison, other regions reported by Arora et al. included Canada, Europe, the United Kingdom (UK) and the United States of America (USA), where 32-61% of endocrinologists were female and 21-44% of invited speakers at endocrinology conferences were female. In all regions other than the UK there was a lower proportion of female speakers than the proportion of female endocrinologists in that location. Arora et al. also noted underrepresentation of females as panellists at conferences and found a strong positive correlation between the proportion of women on the planning committees and proportion of invited female speakers. Perhaps the proportion of females on the organising committees of the conferences we have reported on positively influenced the ratios of female speakers.

We did note an increase in the proportion of female presentation time in symposiums and plenary sessions from 2016 to 2020. It could be anticipated that given the increasing proportion of female trainees and younger average age of female versus male endocrine consultants in Australia, that it is likely that in future years more invited speaker roles will have female representation. However, this was not supported by Gamage et al, who found that in the 7 year period (2013-2019) there was no change in the median proportion of female invited speakers in endocrinology conferences in North America and Europe (6).

Of interest, Gamage et al. also assessed the number of speakers who gave more than one presentation at endocrinology conferences during their 7 years observation period (6). They found that it was more frequent for males to be invited to present at multiple conferences. They highlighted the issue of a lack of diversity of speakers and the risk of the opinions of a smaller group being repeatedly promoted. A lack of diversity limits the development of ideas, and increased diversity should be promoted not only to elevate the underrepresented groups but also for the overall advancement of scientific research (14, 15).

We found that there was an equal representation of females and males as sessions chairs at endocrinology conferences. We did not assess the number of male only chaired sessions, however Salem et al. identified that 47% of sessions were male-only chaired at an endocrinology conference in the UK (16). Engagement in medical conferences at all levels is beneficial for education, networking and sharing of knowledge. Salem et al. assessed engagement of conference delegates at endocrinology conferences in the UK over two years (16). Females asked fewer questions, and questions and comments from men were longer than from women. After an intervention which involved e-mailing organising committees and delegates, there was an improvement in the proportion of sessions chaired by at least one woman and the proportion of questions asked by females. This is an example of the role of increasing awareness of gender equity at conferences with positive outcomes.

We found that women were underrepresented as council members on the diabetes and endocrinology societies in Australia. These findings are supported by Waseem et al. who found that 31.3% of board members of diabetes and endocrinology societies worldwide were female (10). They also showed that female board members were less likely to have higher-faculty rank (Professor, Associate Professor and Assistant Professor) compared to male board members. Similarly there are less women on editorial boards of scientific journals in endocrinology, with women representing 20% of editors in chief and 31.6% of all associate editors (9). It is likely that the underrepresentation of women in these prestigious roles is reinforced by the underrepresentation of women in academic medicine (5).

Professional scientific societies play a crucial role in advancing women in science (17). Medical societies are designed to support their members and their leadership should reflect their membership base. We feel that females should be represented equally at all society activities, including leadership roles. Having appropriate female academic role models and mentors is an important factor in addressing gender disparities (4, 11, 18). Setting targets is useful, however a systematic review identified that organisational interventions are vital (11). In order to overcome established barriers, the suggested interventions included organisational processes such as addressing structural barriers with flexibility to take into account family commitments, as well as promoting awareness and engagement. Mentoring, networking, and leadership development have been identified as key interventions, and scientific societies and their conferences should be facilitating this. Alternate strategies include gender equity programs such as Athena SWAN initiatives, which have had varying success (19).

Scientific societies should be making concerted strategic efforts to build diversity at scientific conferences that would help address the disparities of female representation. The Alliance to Catalyze Change for Equity in STEM Success (ACCESS) is a meta-organisation that has published recommended strategies for fostering inclusivity through speaker diversity in scientific meetings (20). One such recommendation includes collection and publication of demographic information on membership and abstract submission authors in order to ensure speaker profiles reflect membership base. Other recommendations include open calls to membership for participation on speaker selection committees, allowing adequate time for conference programming to ensure inclusivity of speakers, establishing diversity and inclusivity committees which assist with selection of speakers, and the use of speaker referral databases that list qualified speakers from underrepresented groups.

This is the first study to assess the representation of females in a broad range of speaking and leadership roles within endocrinology societies in Australia over a 5-year period. However, the single specialty focus and geographic focus limits the generalisability of the findings. This study did not explore factors that might be associated with gender disparities at conferences or in committee appointments. The female proportion of the membership of each society and the attendance at conferences was not publicly available for inclusion in this paper.

In conclusion, we were pleased to find that within the field of endocrinology in Australia, there is an overall equal representation of female and male speakers at conferences. However, we highlight the ongoing issue of underrepresentation of women as plenary speakers and in more prestigious roles in our diabetes and endocrine societies and implore conscious effort to address this disparity.

## Data Availability Statement

The raw data supporting the conclusions of this article will be made available by the authors, without undue reservation.

## Author Contributions

LR and AM conceived of the presented idea. LR collected the data. Both authors discussed the results and contributed to the final manuscript. All authors contributed to the article and approved the submitted version.

## Conflict of Interest

AM is the President-elect of the Endocrine Society of Australia.

The remaining author declares that the research was conducted in the absence of any commercial or financial relationships that could be construed as a potential conflict of interest.

## Publisher’s Note

All claims expressed in this article are solely those of the authors and do not necessarily represent those of their affiliated organizations, or those of the publisher, the editors and the reviewers. Any product that may be evaluated in this article, or claim that may be made by its manufacturer, is not guaranteed or endorsed by the publisher.
